# GPE Promotes the Proliferation and Migration of Mouse Embryonic Neural Stem Cells and Their Progeny In Vitro

**DOI:** 10.3390/ijms18061280

**Published:** 2017-06-16

**Authors:** Cristina Almengló, Pablo Devesa, Jesús Devesa, Víctor M. Arce

**Affiliations:** 1 Department of Physiology, University of Santiago de Compostela, Santiago de Compostela 15710, Spain; cristina.almenglo@usc.es (C.A.); victor.arce@usc.es (V.M.A.); 2 Research and Development, Medical Center Foltra, Teo 15886, Spain; pdevesap@foltra.org; 3 Scientific Direction, Medical Center Foltra, Teo 15886, Spain

**Keywords:** GPE, GH, neural regeneration, ERK, PI3K/Akt, *N*-methyl-d-aspartate (NMDA), neural stem cells, brain injury

## Abstract

This study was designed to investigate a possible role of the N-terminal tripeptide of insulin-like growth factor-1 (IGF-I), Gly-Pro-Glu (GPE), physiologically generated in neurons following IGF-I-specific cleavage, in promoting neural regeneration after an injury. Primary cultures of mouse neural stem cells (NSCs), obtained from 13.5 Days post-conception (dpc) mouse embryos, were challenged with either GPE, growth hormone (GH), or GPE + GH and the effects on cell proliferation, migration, and survival were evaluated both under basal conditions and in response to a wound healing assay. The cellular pathways activated by GPE were also investigated by using specific chemical inhibitors. The results of the study indicate that GPE treatment promotes the proliferation and the migration of neural stem cells in vitro through a mechanism that involves the activation of extracellular signal-regulated kinase (ERK) and phosphoinositide 3-kinase PI3K-Akt pathways. Intriguingly, both GPE effects and the signaling pathways activated were similar to those observed after GH treatment. Based upon the results obtained from this study, GPE, as well as GH, may be useful in promoting neural protection and/or regeneration after an injury.

## 1. Introduction

Besides its role as a critical mediator of body growth and development [1,2], the insulin-like growth factor-1 (IGF-1) plays also a prominent role in central nervous system (CNS) development and metabolism. IGF-1 is necessary for the normal development of the brain, as revealed by the striking CNS phenotype observed in both IGF-1^−/−^ mice and transgenic mice overexpressing IGF-1 [3]. In the adult life, IGF-1 continues to exert a prominent role in regulating many CNS functions including neuromodulation, neuroprotection, neural plasticity, and neural repair [4,5,6,7]. Owing to these actions, IGF-1 has been proposed as a novel therapeutic in several neurological disorders, although its clinical application is limited by its large molecular size, poor central uptake, and mitogenic potential.

Within the brain, IGF-1 is proteolytically processed by a specific acid protease, generating des-N-(1-3)-IGF-1 (des-IGF-1), and an N-terminal Gly-Pro-Glu tripeptide (GPE) [8,9,10]. Although GPE was initially considered to be a non-bioactive by-product of IGF-1, it was later found that this small peptide was able to significantly increase the release of ACh from rat cortical slices [11,12]. Consequently, several neuroprotective effects of GPE have been unveiled since that discovery (see [13,14,15] for review). Thus, GPE has been shown to protect hippocampal neurons from NMDA-induced toxicity in vitro [11], and to reduce brain injury after hypoxia-ischemia [16,17,18]. In addition, GPE has been found to exert neuroprotective actions in animal models of neurodegenerative processes, such as Huntington’s, Parkinson’s, or Alzheimer’s diseases [19,20,21]. Finally, GPE shows an anti-depressant activity in lipopolysaccharide (LPS)-induced depression in mice [22].

However, the possibility that GPE would also be involved in the regulation of the proliferation of neural stem cells (NSCs) and, therefore, have a potential role in neural regeneration has not been described so far. In this study, we have investigated the effect of GPE on the regulation of the proliferation and survival of mouse embryonic NSCs in vitro. For comparison purposes, we also analyzed the effects of growth hormone (GH) on these cultured cells, because in previous studies we showed that exogenous GH cooperates with locally-produced GH in increasing the proliferation of hippocampal neural stem cells after an injury [23], and we established the signaling pathways by which GH leads to cell proliferation and survival in hippocampal neural precursors [24]. Moreover, the positive effects of GH on brain are well known [25].

Our results demonstrate that GPE exerts a positive effect on the proliferation and survival of NSCs that is mediated, at least in part, via activation of the extracellular signal-regulated kinase (ERK) and phosphoinositide 3-kinase (PI3K)-Akt pathways.

## 2. Results

### 2.1. Neural Differentiation In Vitro

**Figure 1:**
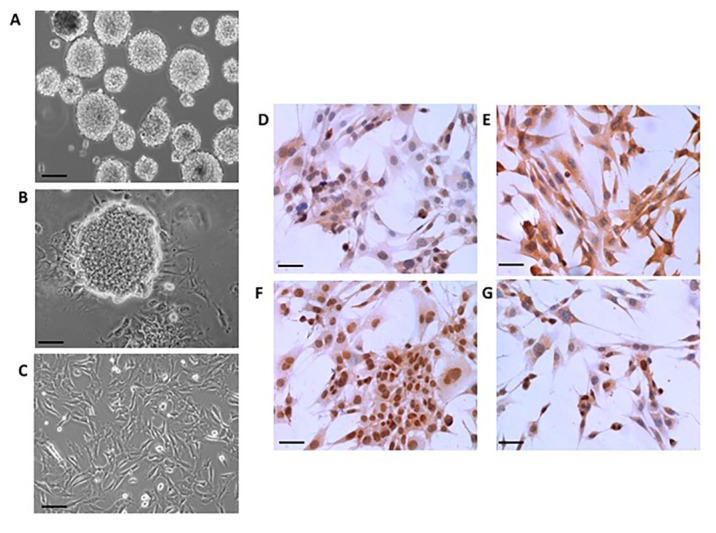
Characterization of mouse embryonic neural stem cell (NSC) cultures. Cells obtained from 13.5 dpc (days post conception) mouse embryo brains were placed into glass coverslips and cultured as indicated. Growing the cells in the presence of semi-synthetic medium containing epidermal growth factor (EGF) and basic fibroblast growth factor (bFGF) resulted in the formation of neurospheres (**A**) that could be differentiated to monolayer cultures after withdrawn of the factors (**B**,**C**); Immunoreactivity against specific markers of the three neural lineages (i.e., neurons, astrocytes, and oligodendrocytes) was revealed in the differentiated cells by using specific antibodies against anti-glial fibrillary acidic protein (GFAP) (**D**); myelin basic protein (MBP) (**E**); and NeuN (**F**); Notice that a significant number of cells retain anti-SOX2 immunoreactivity (**G**); Scale bars 100 µm (**A**,**C**–**F**) or 50 µm (**B**).

Depending on the culture conditions, cells isolated from 13.5 dpc (days post conception) mouse embryo brains can give rise to new neurospheres upon passaging or differentiate into cells expressing characteristic markers of the three neural cell types, i.e., neurons, astrocytes, and oligodendrocytes cells (Figure 1). In addition, a significant number of cells express SOX2, a member of the SoxB1 transcription factor family that is expressed in neural progenitor populations throughout development and is necessary to maintain a neural progenitor state [26]. Altogether, these findings reveal the potential for multi-lineage differentiation of neural cells isolated from 13.5 dpc mouse embryos, and validates their NSC phenotype.

### 2.2. Cell Proliferation Induced by Gly-Pro-Glu (GPE) or Growth Hormone (GH)

**Figure 2:**
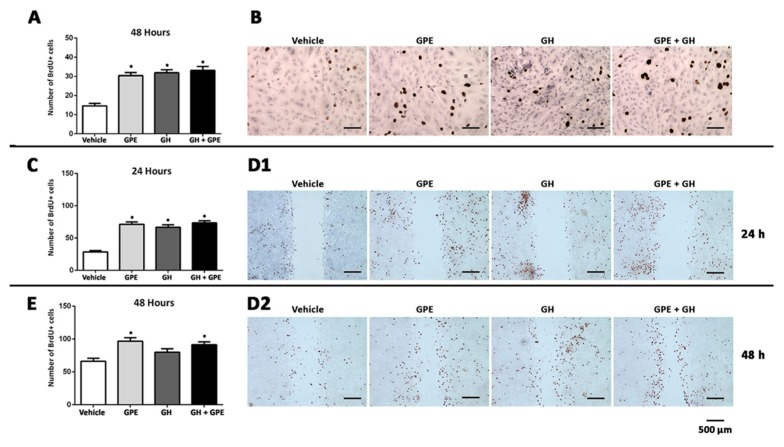
Both Gly-Pro-Glu (GPE) and growth hormone (GH) treatment enhance cell proliferation. (**A**,**B**) Cells were serum-starved for 48 h and then treated with GPE (100 µM), GH (500 ng/mL), or GPE + GH every 24 h for 2 days. Control cells were treated with vehicle. (**A**) Each bar represents the mean + SEM (Standard Error of the Mean) of two independent experiments (*n* = 20); (**B**) Representative images of results are shown in panel **A**; (**C**–**D2**) Cells were serum-starved for 48 h and a scratch wound healing assay was performed (see methods for further details); (**C**,**E**) Each bar represents the mean + SEM of three independent experiments (*n* = 30); (**D1**,**D2**) Representative images of results are shown in panels (**C**,**E**) after 24 h (**D1**) and 48 h (**D2**) of treatments with vehicle, GPE, GH, and GPE + GH (same doses as those described above). * *p* < 0.05 vs. vehicle. Scale bars 500 µm.

Treatment of primary NSC cultures with 100 µM GPE induced a significant increase in the number of BrdU+ cells (Figure 2A,B). This effect was similar to that observed when cells were treated with GH, however, surprisingly, co-treatment with GPE + GH did not elicit any further increase in the number of proliferating cells as compared with the isolated effect of each of them (Figure 2A,B). Similar results were obtained when cell proliferation was determined in a wound-healing assay (Figure 2C–D2). Also in this case, treatment with either GPE or GH induced a significant increase in the number of BrdU+ cells present at the edges of the wound, but further increases in the number of BrdU+ cells were not observed when both hormones were given simultaneously.

### 2.3. Cell Migration Induced by GPE or GH

**Figure 3:**
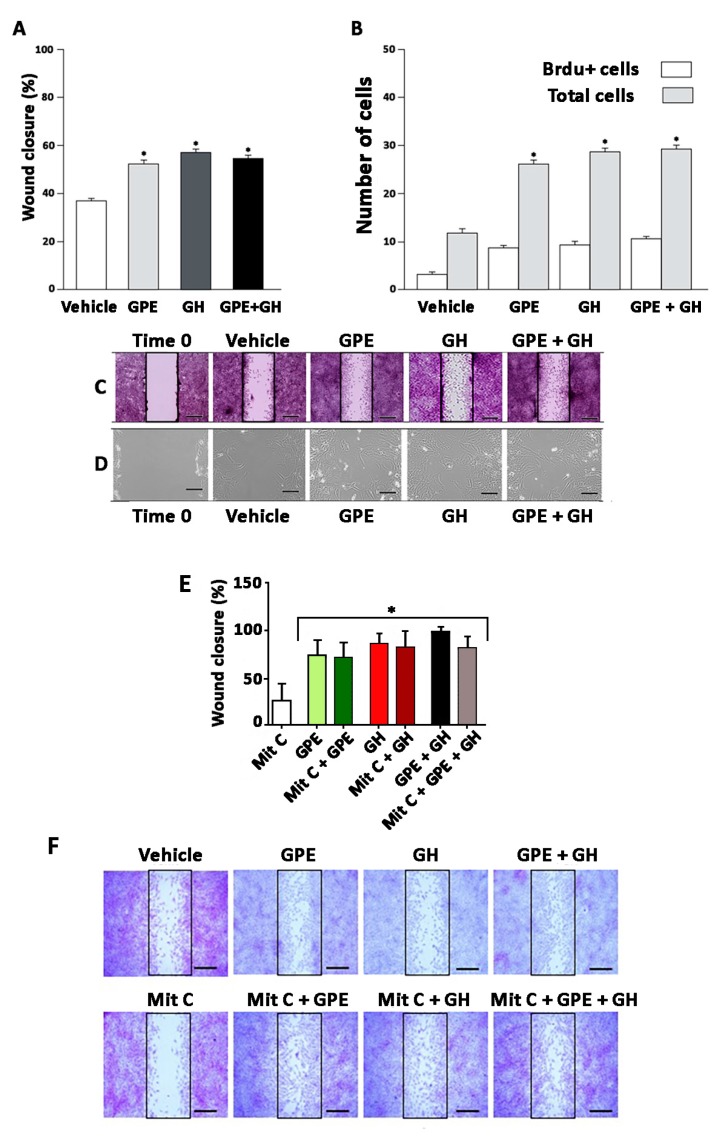
Both GPE and GH stimulate cell migration in scratch wound-healing assay. (**A**,**B**) Cells were serum-starved and treated with GPE (100 µM), GH (500 ng/mL), or GPE + GH every 24 h for 2 days. Control cells were treated with vehicle. (**A**) Percentage of wound closure after 48 h of treatment. * *p* < 0.05 vs. vehicle; (**B**) Total number of cells and cells BrdU+ inside the lesion after 48 h of treatment. * *p* < 0.05 vs. vehicle. Results are representative of three independent experiments (*n* = 30); (**C**,**D**) Representative images of results are shown in panels (**A**,**B**); (**E**) Cells were serum-starved and treated as indicated above in the presence of mitomycin C or vehicle, and the total number of cells inside the lesion was determined after 48 h. Results are representative of three independent experiments (*n* = 30). * *p* < 0.05 vs. basal. Black rectangles in (**C**,**F**) indicate the zone where cells were measured. Images in (**D**) correspond to optical microscopy, without staining. Time 0 in (**C**,**D**) shows the basal situation, before commencing any treatment. Mit C: Mitomycin C. Scale bars 500 µm (**C**) and 50 µm (**D**).

Besides to being useful for studying cell proliferation, the wound-healing assay is a straightforward method to study cell mobility in vitro [27]. As depicted in Figure 3A–D, the creation of a scratch on the confluent cell monolayer stimulated the migration of the cells on the edge of the gap towards the opening with a clear mesenchymal behavior. This migration was significantly increased by either GPE or GH treatment, since both the wound closure and the number of cells present in the gap were higher in either GPE-treated or GH-treated cells as compared with vehicle-treated cells. However, also in this case, the combined administration of GPE + GH did not induce a greater effect that any isolated treatment. Interestingly, despite the increase in the number of cells present in the gap induced by the treatments, no differences were found in the number of BrdU+ cells among the four experimental groups, thus indicating that filling the gap mainly depends on migrating rather than proliferating cells (Figure 3B). To further ascertain this possibility, a wound healing assay was performed in the presence of mitomycin C, a chemotherapeutic antibiotic that, reportedly, inhibits cellular growth [28]. In keeping with our hypothesis, inhibition of cell proliferation did not significantly modify the effect of either GPE or GH treatment (or their combination) on the wound closure (Figure 3E,F).

### 2.4. GPE Signaling Pathways

**Figure 4:**
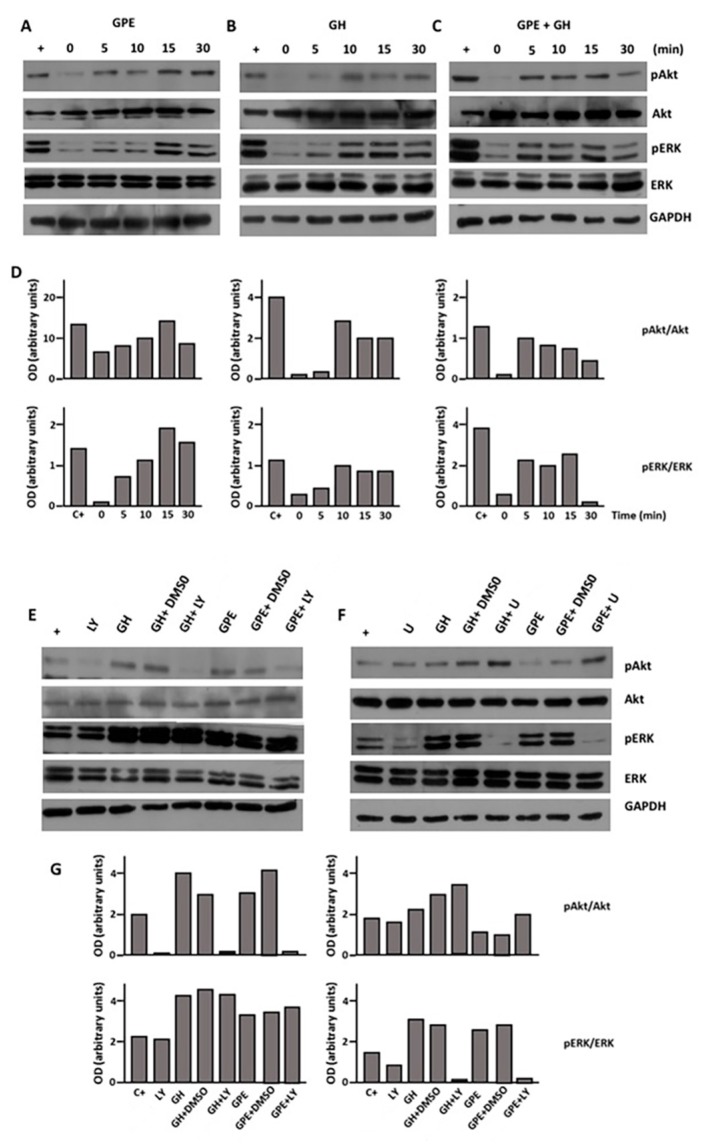
Both GPE and GH stimulate Akt and extracellular signal-regulated kinase (ERK) phosphorylation. Cells were serum-starved and treated with GPE (100 µM), GH (500 ng/mL), or GPE + GH for the indicated times. (**A**–**C**) Akt, phospho-Akt, ERK, phospho-ERK, and glyceraldehyde 3-phosphate dehydrogenase (GAPDH) immunoreactivities were determined by western blot; (**D**) Densitometric analysis of results presented in panels (**A**,**C**). Results are shown as the pAkt/Akt (**upper**) or pERK/ERK ratio (**lower**); (**E**,**F**) Akt and ERK phosphorylation were investigated in the presence of the specific inhibitors LY294002 (LY) and U0126 (U). C+ indicates the positive control (not serum-starved cells); (**G**) Densitometric analysis of results presented in panels **A** to **C**. Results are shown as the pAkt/Akt (**upper**) or pERK/ERK ratio (**lower**).

Once we demonstrated these biological effects of GPE on NSCs, we intended to delineate their mechanisms of action. To this end, we investigated two signaling pathways classically involved in the control of cell proliferation, such as the PI3K-Akt and ERK pathways. As shown in Figure 4, treatment of monolayer cultures of NSCs with GPE induced a prompt rise in the levels of phosphorylated Akt. This effect was observed as soon as 5 min after the treatment, with the maximal activation observed at time 15 min (Figure 4A). In contrast, when cells were treated with GPE in the presence of LY294002, a selective inhibitor of PI3K, Akt phosphorylation was completely prevented (Figure 4D). GPE treatment also induced the phosphorylation of Akt, with a similar pattern of activation (Figure 4A). Also in this case, GPE-induced phosphorylation was abrogated when GPE was given in the presence of U0126, a highly selective inhibitor of both MEK1 and MEK2 (Figure 4E).

Similar results were obtained when cells were treated with GH (Figure 4B). In keeping with previous reports in hippocampal neural precursors [24], GH elicited the activation of both PI3K-Akt and ERK signaling pathways (Figure 4B) that was prevented using selective inhibitors (Figure 4D,E). As also occurred when we investigated the effect of combined administration of GPE and GH on cell proliferation and migration, no differences were observed on either Akt or ERK phosphorylation when both drugs were given simultaneously, as compared with their independent effects (Figure 4C). Activation of either Akt or ERK signaling induced by GPE + GH was also prevented in the presence of specific inhibitors (Figure 4D,E).

### 2.5. The Role of ERK and Akt on Cell Proliferation and Migration

**Figure 5:**
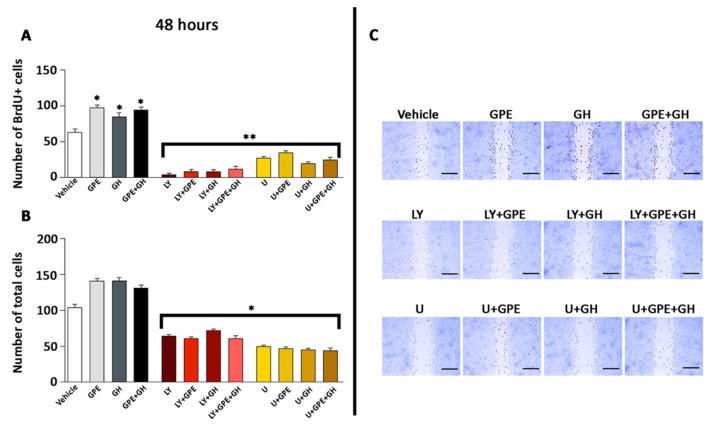
The PI3K/Akt and ERK pathways are involved in the proliferative effect of both GPE and GH. Cells were serum-starved for 48 h and a scratch wound healing assay was performed (see methods for further details). Cells were then treated for 24 h with GPE (100 µM), GH (500 = ng/mL), or GPE+GH, in the presence of LY294002, U0126, or vehicle. BrdU (10 mM) was given 2.5 h before the end of the treatments and BrdU+ cells were detected by immunocytochemistry. (**A**) Number of BrdU+ cells at the edge of the wound and within the gap. Each bar represents the mean + SEM of two independent experiments (*n* = 20). * *p* < 0.05 vs. vehicle; ** *p* < 0.01 vs. vehicle, GPE, GH, and GPE + GH; (**B**) Total cells (BrdU+ and BrdU−) present within the gap. Each bar represents the mean + SEM of two independent experiments (*n* = 20). * *p* < 0.05 vs. vehicle; (**C**) Representative images of data shown in panels (**A**,**B**). LY = LY294002. U = U0126. Scale bars 500 µm.

To gain further evidence about the role of Akt and ERK signaling pathways on GPE effects on NSCs, we envisaged the effect of the blockade of these paths on cell proliferation by using the wound-healing assay (Figure 5). As indicated above, GPE, GH, or GPE + GH treatment significantly increased the number of BrdU+ cells present at the edges of the wound. However, this effect was dramatically decreased in all cases by treating the cells with either LY294002 or U0126 (Figure 5A,B). Similar results were obtained when the number of cells filling the gap was investigated. All treatments increased the number of cells observed within the gap, an affect that was thwarted by using the specific inhibitors (Figure 5C). Overall, these findings suggest that both ERK and Akt are involved in the proliferation and migration induced by either GPE or GH treatment in NSCs.

### 2.6. The Role of ERK and Akt on Cell Survival

**Figure 6:**
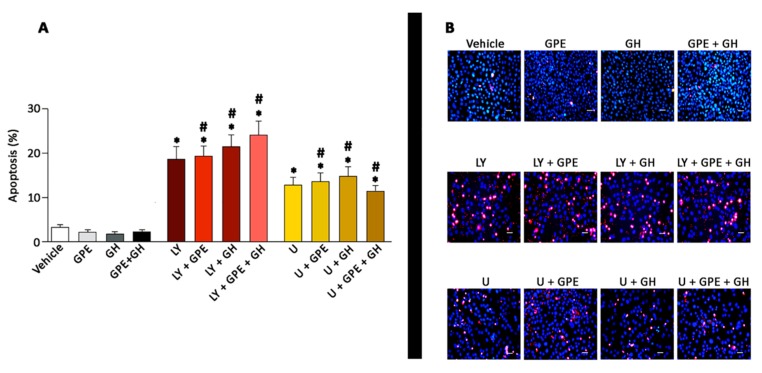
Blockade of PI3K K/Akt or ERK pathways results in activation of apoptotic cell death of NSCs that is not counteracted by either GPE or GH treatment. Cells were seeded in 24-well plates and treated with GPE (100 µM), GH (500 ng/mL), GPE + GH (100 µM and 500 ng/mL, respectively) or vehicle every 8 h for 1 day, in the presence of LY294002, U0126 or dimethyl sulfoxide (DMSO) (vehicle). Cells were stained with Hoestch 33,258 (1 µg/µL) and propidium iodide (1 µg/µL), and cell morphology was examined under a fluorescence microscope. (**A**) Each bar represents the mean + SEM of three different experiments in triplicate. * *p* < 0.05 vs. vehicle, # *p* < 0.05 vs. respective control (i.e., LY+GPE vs. GPE, etc.); (**B**) Representative images of results are shown in panel A. Scale bars 500 µm.

The involvement of Akt and ERK signaling on GPE actions was further explored by investigating their role on cell survival. As Figure 6 depicts, either GPE or GH treatment, as well as their combination sharply, reduced the number of apoptotic cells as compared with controls, although no statistical significance was achieved, probably because of the low number of apoptotic cells existing under these experimental conditions. Pharmacological blockade of PI3K/Akt signaling resulted in a significant 4-fold increase in the number of apoptotic cells, that was not counteracted by any of the treatments tested (GPE, GH, or GPE + GH). Similarly, the blockade of the ERK pathway with U0126 induced a 3-fold significant increase in the number of apoptotic cells. Also in this case, stimulation of apoptotic cell death was not modified by either GPE or GH or their combination. In all, these results reinforce the role of Akt and ERK in both GPE and GH signaling.

### 2.7. Blockade of the NMDA Receptor and GPE Signaling Pathways

**Figure 7:**
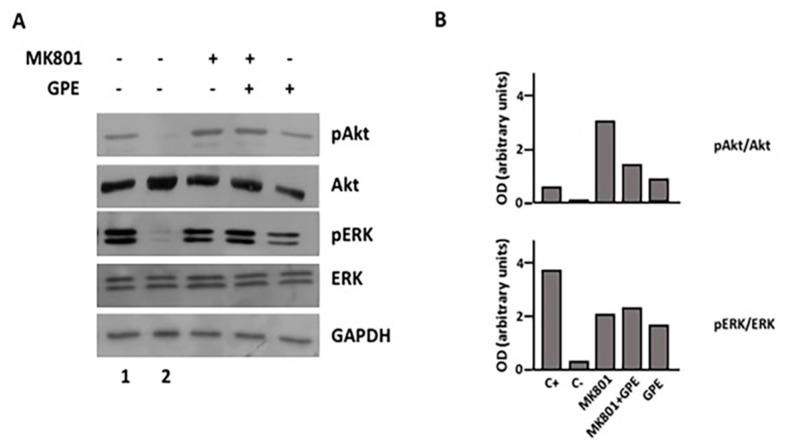
Blockade of NMDA receptors promotes Akt and ERK phosphorylation. (**A**) Cells were serum-starved for 48 h and then treated with MK-801 (100 µM, +) or vehicle (-) for 2 h. At the end of the treatment, cells were challenged during 15 min with GPE (100 µM, +) or vehicle (-). Serum-treated and serum-starved (48 h) cells were used as positive (**1**) and negative (**2**) controls, respectively; (**B**) Densitometric analysis of results are presented in panel **A**. Results are shown as the pAkt/Akt (**upper**) or pERK/ERK ratio (**lower**).

Finally, since the mechanism underlying the actions of GPE remains unclear, we investigated the possible involvement of the NMDA receptor, one of the proposed mechanisms by which GPE might act, by using a selective inhibitor of NMDA activation. Reportedly, GPE interacts with NMDA receptor, but both antagonist and weak-agonist effects have been described. As Figure 7 depicts, GPE treatment promotes the phosphorylation of both Akt and ERK in the presence of MK801. Furthermore, blockade of NMDA receptor by MK801 treatment also induced a sharp increase in the phosphorylation of both Akt and ERK, similar to that observed after GPE treatment. Overall, these results suggest that the effects GPE effects may also be mediated by its ability to bind and blockade the NMDA receptor.

## 3. Discussion

Several reports have demonstrated that GPE exerts a neuroprotective effect in both in vitro and in vivo systems. Thus, administration of GPE protects neurons from NMDA-induced toxicity rat cortical slices, while central administration of GPE reduces the extent of cortical infarction in several models of hypoxia-ischemia in rats [15,16,17,18,29]. GPE has also been proved to be effective in preventing some of the clinical outcomes present in several animal models of degenerative diseases and, more recently, to exert an anti-depressant activity in mice [19,20,21,22]. In this study, we have described a novel effect of GPE in neural cells, such as the stimulation of cell proliferation and, to some extent, cell migration. Altogether, these findings would support a role for GPE not only in neuroprotection but also in neural repair.

Neural progenitors are cells that are capable of dividing a limited number of times and have the capacity to differentiate into a restricted repertoire of neuronal and glial cell types. Due to these characteristics, neural progenitors may be used to replace the neuronal loss that is the hallmark of several CNS diseases [30]. In this report, we have found that GPE administration promotes the proliferation and, to a lesser extent, the migration of neural progenitors. This effect was observed either in cells growing under basal conditions or as a response to mechanical damage induced by using an in vitro model of wound repair. Interestingly, the results obtained in this study are very similar to those observed when GH, a well-known promoter of neural regeneration, was used under the same experimental conditions. This resemblance comprises both the biological effects and biochemical mechanisms involved. In fact, both compounds yield comparable results on both cell proliferation and migration in all the experimental settings investigated and, intriguingly, no additive effect was observed when given together. Similarly, activation of either ERK or Akt signaling pathways was almost identical for both hormones. This includes both the maximal activation achieved and the time-course of activation observed. Altogether, these equivalent results suggest that GPE and GH are activating the same signaling pathways (namely ERK and PI3K-Akt) to promote cell proliferation and migration. In fact, these signaling mechanisms are among the best characterized in the regulation of cell proliferation, differentiation, and survival in response to a wealth of hormones and growth factors in many cell types [31,32,33]. Therefore, it is tempting to speculate that GPE treatment may be used, as occurs with GH, to promote neural repair in vivo. However, since GPE has an extremely short mean-life in vivo, further studies will be necessary to investigate whether the GPE analogues with longer half-lives that are currently being tested in clinical trials [34] show similar effects on cell proliferation and migration. In addition, the effects of novel IGF-I-derived smaller peptides are now being studied in human patients [35].

Despite the accumulating evidence supporting a role for GPE in CNS protection and repair, the molecular mechanisms underlying these effects remain unclear. Although GPE sequence is essential for IGF-1 binding to IGF-1 binding proteins, it is presently clear that GPE does not interact with IGF-1 receptor [14]. Owing to its glutamate residue, the possibility that GPE could act through the *N*-methyl-d-aspartate (NMDA) receptor has also been raised. In this regard, radioligand-binding studies have suggested that GPE binds to NMDA receptors in Müller glia and activation of NMDA receptor mediates the mitogenic effects of GPE in these cells [36]. In contrast, Sara et al. [11] showed that GPE inhibits glutamate binding to the *N*-methyl-d-aspartate (NMDA) receptor, thus inducing the release of acetylcholine from rat cortical slices and, to a lesser extent, the release of dopamine from striatum. Furthermore, the neuroprotective effect induced by GPE is similar to that of MK801, a noncompetitive NMDA receptor antagonist [11], which has also been proved to promote ERK and Akt phosphorylation in a similar fashion to that of GPE (this study).

On the other side, it has been reported that the prevention of neuronal death showed by GPE is not directly related to its affinity by glutamate receptors [37]. Indeed, GPE has been recently shown to have a very low binding affinity for NMDA, thus making it unlikely that NMDA is its physiological receptor [38]. While one possible explanation for these discrepancies may be differences in the dose of GPE and/or the experimental settings used, it seems to be clear that further experiments are still necessary to unveil the physiological receptor mediating GPE actions.

Although our results do not allow us to determine the nature of the GPE receptor, the fact that we have observed a rapid phosphorylation for both, and different, signaling pathways (ERK and Akt) suggests that the activation of these is a direct effect of receptor stimulation rather than a cross-talking mechanism. At present, there is strong evidence supporting a relationship between NMDA receptor and both ERK and Akt activation in neural cells. Thus, agonist binding of the NMDA receptor increases postsynaptic intracellular [Ca^2+^] and activates the ERK pathway in neurons through a direct interaction of the receptor with Ras protein specific guanine nucleotide releasing factor 1 (RasGRF1) [39,40]. Similarly, activation of Akt has also been reported in cortical neurons through a mechanism that mirrors ERK activation and may also involve Ca^2+^ influx [41]. Altogether, these findings might indicate the possibility that NMDA receptor is also involved in the GPE-induced activation of ERK and Akt signaling in NSCs. However, we also report here that inhibition of the NMDA receptor with a non-competitive agonist results in activation of both signaling pathways in a fashion that resembles that observed after GPE treatment. This result makes it unlikely that GPE is stimulating NMDA receptor to promote ERK and Akt activation (and, therefore, cell proliferation and migration), and suggests that the effect of GPE may depend on NMDA receptor blockade, as previously proposed by Sara et al. [11]. In fact, the existence of a NMDA receptor-mediated inhibitory pathway that functions to attenuate or shut-off ERK and Akt signaling has also been previously reported in neurons [42]. On this basis, it seems conceivable that blockade of this pathway by GPE would result in activation of ERK and Akt signaling. While this NMDA receptor-mediated inhibitory pathway does not appear to predominate in neurons, it has been proposed to be selectively coupled to activation of extrasynaptic NMDA receptors. Since our studies are not performed in neurons but in neural progenitors, it is possible to speculate that, at odds with neurons, NMDA receptor-mediated inhibitory pathway is predominating in these cells. Another possibility is that GPE actions may occur as a consequence of indirect effects on the NMDA receptor. Moreover, since we did not observe any additive effect when cells were treated with GPE + GH, the possibility exists that GPE and GH share a mechanism for activating ERK and Akt through different receptors.

Another possibility, not tested in our study, is that GH might induce the expression of IGF-I in the cells we cultured, and this newly-formed IGF-I could have originated GPE, therefore explaining the similar effects of GH and GPE and the lack of synergism between them. In fact, it has been demonstrated [43] that GH induces the expression of IGF-I in human fetal forebrains. However, the fast effects of both GH and GPE on the phosphorylation of Akt and ERK makes such a possibility quite unlikely.

In summary, our results show that GPE promotes the proliferation and migration of neural progenitors through a mechanism that involves the activation of ERK and PI3K-Akt pathways, thus supporting a possible role for GPE (or any of its analogues) for promoting neural repair in vivo. This possibility may be overestimated because in this study we used embryonic stem cells, whose potential for neural regeneration is quite different to that of adult stem cells. However, some preclinical studies [42,44,45] and clinical trials currently in course indicate that GPE or its derivatives possess important beneficious effects as neuroprotective and neuroregenerative factors.

## 4. Materials and Methods

### 4.1. Cell Cultures and Treatments

Monolayer cultures of mouse embryonic NSCs were established from 13.5 days C57BL/6 mice embryos. The care and use of all experimental animals was in accordance with institutional guidelines (EU Directive 2010/63/EU for animal experiments). It was approved by the Ethical Committee for Animal Research of the Xunta de Galicia (15005AE/11/FIS 02/VMAV/1, 14/October/2011). Briefly, cells were dissociated by mechanical shearing followed by filtration through a 40-micron mesh. Cells were then plated in Falcon polystyrene culture dishes (BD Biosciences, Madrid, Spain) and grown in Dulbecco’s modified Eagle’s medium (DMEM, Sigma-Aldrich, Madrid, Spain) supplemented with 10% fetal bovine serum (FBS) (Thermo Fisher Scientific, Madrid, Spain), 1% l-glutamine, and 1% penicillin/streptomycin (all from Thermo Fisher Scientific). Formation of neurospheres was induced by growing the cells in DMEM supplemented with 1% Gibco B27 supplement, 10 ng/mL epidermal growth factor (EGF), 10 ng/mL basic fibroblast growth factor-2 (bFGF-2) (all from Thermo Fisher Scientific), 100 U/mL penicillin, and 100 μg/mL streptomycin. Development of monolayer cultures from neurospheres was achieved by placing the neurospheres in DMEM supplemented with 10% FBS, 1% l-glutamine, and 1% penicillin/streptomycin. All cultures were maintained at 37 °C in a humidified atmosphere of 5% CO_2_. Note that these cultures were composed of a mixed population of progenitor and differentiated neural cells.

Except where indicated, all experiments were performed in cells serum-starved (0% FBS) for 48 h. Cells were then treated with GPE (Bachem AG, Bubendorf, Switzerland, 100 µM), GH (Humatrope, Eli Lilly and Co., Madrid, Spain, 500 ng/mL), or GPE + GH. GPE dose was determined as the minimal dose that was able to induce cell proliferation. GH dose was established according with previous reports [24,46]. Control cells were treated with vehicle. The antimitotic drug mitomycin was purchased from Sigma and used at 5 µM. The PI3K-Akt and ERK signaling pathways inhibitors (LY294002 and U0126, respectively) were purchased from Cell Signaling Technology (Danvers, Werfen/Izasa, Hospitalet de Llobregat, Spain), and used at 10 µM. The selective non-competitive NMDA receptor antagonist, MK-801, was purchased from Sigma-Aldrich and used at 100 µM.

### 4.2. Immunocytochemistry

Approximately 5 × 10^3^ cells were seeded in glass coverslips, placed in 24-multiwell plates, and incubated for 24 h as described above. Cells were then fixed in 96% ethanol for 1 h, rinsed in posphate-buffered saline (PBS), and permeabilized by adding 250 μL of 0.1% Triton X-100, 0.3% bovine serum albumin (BSA) for 1 h. After rinsing the cells three times in PBS, an overnight incubation at 4 °C was performed with the following primary antibodies: anti-glial fibrillary acidic protein (GFAP) (polyclonal, Dako, Agilent Technologies, Madrid, Spain; ready to use), anti-myelin basic protein (MBP) (polyclonal, Dako, Agilent Technologies; dilution 1:2000), anti-NeuN (monoclonal, Merk Millipore, Madrid, Spain; dilution 1:200), and anti-SOX2 (polyclonal, Seven Hills Bioreagents, Cincinnati, OH, USA; dilution 1:1000). Positive cells were detected with a universal second antibody kit that uses a peroxidase-conjugated labeled-dextran polymer (Envision Plus, Dako, Agilent Technologies, Santa Clara, CA, USA) and photographed with an Olympus BX43 camera (Olympus Optical Co., Tokyo, Japan).

### 4.3. Cell Proliferation Assays

Cells were seeded at 10^6^ cells/mL in poly-l-lysine-coated slides (1 mL/slide) and maintained for 24 h. Cells were the serum-starved for 48 h before being treated with GPE, GH, or GPE + GH every 24 h for 2 days. Control cells were treated with vehicle. BrdU (10 mM, Sigma-Aldrich) was given 45.5 h after the beginning of the treatments and maintained for 2.5 h. BrdU+ cells were detected by immunocytochemistry using a monoclonal antibody (BD Biosciences, dilution 1:2000). Representative images were captured with an Olympus DP72 camera, and the number of BrdU+ cells was quantified in 10 random fields per slide.

### 4.4. Wound-Healing Assays

The wound healing process entails the migration and proliferation of different cell types in vitro. To perform the wound-healing assay, cells were seeded at approximately 1 × 10^5^ per slide and incubated as previously described. Using a plastic pipette tip, a wound field was created with a defined gap of 1 mm, and cells were then treated with GPE, GH, GPE + GH, or vehicle, as indicated above. BrdU+ cells were detected by immunocytochemistry, as previously indicated, and random fields were photographed with an Olympus DP72 camera. The number of cells (total and BrdU+) within the gap and at the edge of the lesion was quantified with the image J software (https://imagej.nih.gov/ij/index.html). In addition, the area of the lesion was also evaluated as previously reported by Latifi-Pupovciet et al. [47].

### 4.5. Western Blot

For phospho-ERK 1/2 and phospho-Akt determination, cells were serum-starved and treated as described. Cells were then collected and lysed by adding RIPA buffer with sodium orthovanadate (10 µL/mL, Sigma), Phenylmethylsulfonyl fluoride (PMSF) (10 µL/mL), aprotinin (20 µL/mL), and NaF 50 mM (all from Sigma). Cell extracts were resolved on 10% sodium dodecyl sulfate polyacrylamide gel electrophoresis (SDS-PAGE) and electro blotted onto a nitrocellulose membrane (Trans-Blot Transfer Medium 0.2 µm, BioRad Laboratories, Madrid, Spain). Primary antibodies against Akt (dilution 1:1000), phospho Akt (dilution 1:1000), ERK 1/2 (dilution 1:10,000), phospho ERK 1/2 (dilution 1:1000) (all from Cell Signaling Technology), and glyceraldehyde 3-phosphate dehydrogenase (GAPDH) (Santa Cruz Biotechnology, Heidelberg, Germany; dilution 1:5000) were applied overnight at 4 °C or 4 h at room temperature. Immunoreactive bands were detected with a western-light chemiluminescence detection system (ECL, APBiotech, Little Chalfont, UK) and photographed (HyperfilmECL, APBiotech, IBM, Madrid, Spain). Band density was evaluated with the Image J software.

### 4.6. Determination of Cell Apoptosis

Apoptotic cell death was determined by nuclear staining with Hoestch 33258 (1 µg/µL, Thermo Fisher Scientific) and propidium iodide (Merk Millipore, 1 µg/µL). Briefly, cells were seeded in 24-multiwell plates and treated with GPE (100 µM), GH (500 ng/mL), GPE + GH (100 µM + 500 ng/mL), or vehicle every 8 h for 1 day, in the presence of LY294002, U0126, or DMSO (vehicle). Twenty minutes before the end of the treatment period cells were stained with Hoestch 33,258 and propidium iodide and, after rinsing, cell morphology was examined under an Olympus fluorescence microscope (TH4-200) with the appropriate filter combination. Cells were scored for apoptosis by their nuclear morphology (shrinkage, condensation, and fragmentation) and the higher intensity of blue fluorescence.

### 4.7. Statistical Analysis

Statistical analysis was performed with one-way ANOVA and Dunnett´s multiple comparisons test. Statistical significance was established at *p* < 0.05.
